# Self-Assembling Nanoarchitectonics of Twisted Nanofibers of Fluorescent Amphiphiles as Chemo-Resistive Sensor for Methanol Detection

**DOI:** 10.3390/gels9060442

**Published:** 2023-05-26

**Authors:** Vandana Singh, Ayyapillai Thamizhanban, Krishnamoorthy Lalitha, Dinesh Kumar Subbiah, Arun Kumar Rachamalla, Vara Prasad Rebaka, Tohira Banoo, Yogendra Kumar, Vellaisamy Sridharan, Asrar Ahmad, Uma Maheswari Chockalingam, John Bosco Balaguru Rayappan, Azmat Ali Khan, Subbiah Nagarajan

**Affiliations:** 1 Department of Chemistry, School of Chemical and Biotechnology, SASTRA Deemed University, Thanjavur 613401, Tamil Nadu, India; vandanarewa@gmail.com (V.S.); umamaheswaric@scbt.sastra.edu (U.M.C.); 2 Centre for Nano Technology & Advanced Biomaterials (CeNTAB), School of Electrical & Electronics Engineering, SASTRA Deemed University, Thanjavur 613401, Tamil Nadu, Indiaboscog@gmail.com (J.B.B.R.); 3 Assembled Organic and Hybrid Materials Research Laboratory, Department of Chemistry, National Institute of Technology Warangal, Warangal 506004, Telangana, India; 4 Department of Chemistry and Chemical Sciences, Central University of Jammu, Rahya-Suchani (Bagla), District-Samba, Jammu 181143, Jammu and Kashmir, India; vesridharan@gmail.com; 5 Center for Sickle Cell Disease, College of Medicine, Howard University, Washington, DC 20001, USA; 6 Pharmaceutical Biotechnology Laboratory, Department of Pharmaceutical Chemistry, College of Pharmacy, King Saud University, Riyadh 11451, Saudi Arabia

**Keywords:** gels, molecular self-assembly, amphiphiles, gelators, sensor, methanol detection

## Abstract

The inhalation, ingestion, and body absorption of noxious gases lead to severe tissue damage, ophthalmological issues, and neurodegenerative disorders; death may even occur when recognized too late. In particular, methanol gas present in traces can cause blindness, non-reversible organ failure, and even death. Even though ample materials are available for the detection of methanol in other alcoholic analogs at ppm level, their scope is very limited because of the use of either toxic or expensive raw materials or tedious fabrication procedures. In this paper, we report on a simple synthesis of fluorescent amphiphiles achieved using a starting material derived from renewable resources, this material being methyl ricinoleate in good yields. The newly synthesized bio-based amphiphiles were prone to form a gel in a broad range of solvents. The morphology of the gel and the molecular-level interaction involved in the self-assembly process were thoroughly investigated. Rheological studies were carried out to probe the stability, thermal processability, and thixotropic behavior. In order to evaluate the potential application of the self-assembled gel in the field of sensors, we performed sensor measurements. Interestingly, the twisted fibers derived from the molecular assembly could be able to display a stable and selective response towards methanol. We believe that the bottom-up assembled system holds great promise in the environmental, healthcare, medicine, and biological fields.

## 1. Introduction

Globally, the emission of volatile organic compounds (VOC) during various chemical processes is considered to be the foremost risk necessitating the periodic monitoring of air quality at a low concentration level, especially in industries. Rapid urbanization and industrialization in the current scenario are generally accompanied by the discharge of VOCs, including hydrocarbons, alcohol, and ketones, to name a few, which could contribute significantly to air pollution, deliberately [1]. Consequently, several adverse effects on human health, retarded plant growth, and ecosystem disturbances are observed [1]. Though instrumental methods of chemical analysis using Fourier transform infrared (FTIR), non-dispersion IR (NDIR), tunable diode laser absorption spectroscopy (TDLAS), differential optical absorption spectroscopy (DOAS), and gas chromatography are readily available to sense and monitor the VOC level, their usage is very limited because of the lack of portability, extensive analysis period, and sample preparation for the VOC analysis [2,3,4,5]. These limitations paved the way for the development of sensors that employ conducting polymers, semi-conducting metal oxide, carbonaceous materials, and hybrid nanomaterials to detect VOCs [3,6,7,8,9]. However, the use of hybrid nanomaterials for sensing VOCs encountered problems, such as toxicity, complex fabrication steps, poor selectivity, interference from non-desirable analytes, and low thermal stability [3]. Especially in this plastic era, the extensive use of crude oil fraction in various chemical industries leads to severe VOC emissions. Hence, the development of nanoarchitectonics derived from smart functional molecules is necessitated in terms of sensing VOCs. Inspired by the natural assembly process, several stimuli-responsive smart materials have been reported in the recent past, with their applications demonstrated in biological, medical, environmental, and other engineering sectors [10,11]. In particular, assembled functional materials derived from organic molecules or metal composites displayed significance in the field of actuators, drug delivery, wound healing, tissue engineering scaffold, sensors, and image enhancement; this involves non-covalent interactions, such as hydrophobic, van der Waals interaction, metal-ligand coordination, H-bonding, and π-π interactions [11]. Among the selection of small molecules for the construction of assembled architectures, the selection of molecules from renewable raw materials is considered a smart move.

In this report, we selected ricinoleic acid obtained from castor oil as a starting material. Generally, castor oil is used as a bio-resource to produce lubricant additives, antioxidants, coatings, surfactants, biodegradable polymers, bio-plasticizers, medicine, and capping/stabilizing agents [12,13,14,15]. Mizota and co-workers revealed the suitability of ricinoleic acid derivative as an electrolyte in capacitors [15]. Recently, our research group has reported on the synthesis and self-assembly of glycolipids derived from ricinoleic acid in a variety of solvents/vegetable oils into oleogels/organogels, which could be used in food, pharmaceutical, and agricultural applications [16]. The traditional use and recent research using castor oil and its derivatives motivated us to choose methyl ricinoleate as one of the raw materials to form amphiphiles, which then underwent supramolecular self-assembly to generate functional architecture. In the field of gas sensors, we demonstrated the practical application of self-assembled carbohydrate-based copper nanoparticles as trimethylamine (TMA) sensors [17]. It is worth mentioning that the flexible fabric material developed in our lab via the interaction of the self-assembled silver-incorporated glycolipids with cellulose fibers displayed fidelity in noxious gas detection [18]. In chemical engineering, a large number of industries and laboratories frequently use methanol as their feedstock or as a solvent; methanol intoxication may create blindness, damage the tissue irreversibly, or even result in death [19]. Gűnter and co-workers reported on the development of a chemo-resistive gas sensor using Pd-doped SnO_2_ nanoparticles to detect methanol selectively within 2 min from 1 to 1000 ppm [19]. In addition to the health and medicine sectors, sensors are very crucial in machinery. Mao et. al. have developed a ZnO-based sensor for the detection of water content in hydraulic oil; the presence of traces of water is one of the reasons for machinery failure in hydraulic systems [20]. A periodical torsion motion hydrogel-derived chemical energy could be used for the development of soft actuators without any external component [21]. Recently, most of the reported methanol sensors have involved the use of either expensive and toxic metals or complicated material fabrication procedures. In this report, we presented a simple synthesis of fluorescent amphiphiles derived from methyl ricinoleate, one of the cheap renewable raw materials, and investigated their self-assembly mechanism, optical and viscoelastic characteristics. The suitability of the self-assembled twisted nanofibers for sensing different analytes, such as 1-butanol, acetone, ethanol, formaldehyde, isopropanol (IPA), methanol, and trimethylamine (TMA), was investigated and the selective sensing response of twisted fibers of **4a** towards methanol over other target analytes was demonstrated. In recent years, the design and synthesis of low-molecular-weight gelators with diverse functional moieties supporting intermolecular interactions have increased significantly due to their aptness in building a reversive supramolecular architecture suitable for a broad range of applications, such as drug carriers, sensors, food formulations, cosmetics, smart devices, and electronics. In line with this, a large library of amphiphilic molecules was generated based on fatty acids derived from vegetable oil that could act as building blocks to construct functional materials [22,23,24]. However, a simple smart self-assembled material that can detect noxious gases is not explored here.

## 2. Results and Discussion

### 2.1. Synthesis

**Scheme 1:**
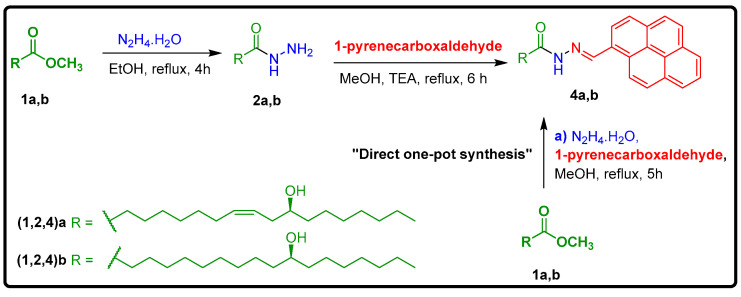
Synthesis of pyrene-appended ricinolyl hydrazone, **4a** and **4b**.

In this report, we used methyl ricinoleate, one of the important renewable raw materials, and 1-pyrenecarboxaldehyde to synthesize fluorescent amphiphiles. To attain a saturated version of methyl ricinoleate **1b**, we reduced the unsaturation present in the methyl ricinoleate using H_2_, Pd/C as a heterogeneous catalyst. The appearance of signals at δ 3.60, 3.55–3.44, 2.34–1.14, and 0.81 ppm in the ^1^H NMR spectrum corresponding to –COOCH_3_, –CH(OH)–, –CH_2_– and –CH_2_CH_3_ protons, respectively, confirmed the formation of **1b** (ESI, Figure S1). The target fluorescent amphiphiles could be obtained either by a two-step or by one-pot synthesis. In the two-step reaction, both saturated and unsaturated esters (**1**) were converted into their corresponding hydrazides (**2**) by reacting with hydrazine hydrate (Scheme 1); then, they were characterized using NMR spectroscopy. The absence of a singlet at δ 3.60 ppm corresponding to –COOCH_3_ and the appearance of broad peaks at δ 6.79 and 3.92 ppm corresponding to –NH, –NH_2_ protons in the ^1^H NMR spectrum of **2a** confirmed the hydrazide formation (ESI, Figure S7). The target amphiphiles (**4a** and **4b**) were synthesized in good yields by refluxing the methanolic solution of hydrazides (**2**) with 1-pyrenecarboxaldehyde in the presence of triethylamine (Scheme 1). The commercially viable one-pot synthesis of **4a** and **4b** in excellent yield was achieved by the direct reaction of the corresponding esters (**1**) with hydrazine hydrate, followed by the condensation of 1-pyrenecarboxaldehyde. This method avoided the use of an organic base, namely, triethylamine. The disappearance of the broad singlet at δ 6.79 ppm corresponding to –NH_2_ and the appearance of the singlet at δ 9.47 and 8.74 ppm corresponding to –NH and –CH=N in the ^1^H NMR spectrum of compound **4a** confirmed the formation of hydrazones.

### 2.2. Gelation Studies

Owing to the immense applications of gels in our day-to-day life, they are considered an attractive soft material displaying tunable properties [25]. Hence, after the synthesis and characterization, we were curious to examine the gelation ability of the newly synthesized fluorescent amphiphiles in various organic solvents/oils using the “stable to inversion method” (Table 1). For the gelation test, we used a broad range of solvents, such as ethylene glycol, glycerol, methanol, lauryl alcohol, polyethylene glycol (PEG), xylene, ethyl acetate, cyclohexane, dichlorobenzene, and water; oils, such as castor, hazelnut, jojoba, linseed, olive, sesame, soybean oil, and paraffin oil were also used. Compounds **4a** and **4b** formed gels in most of the tested oils/solvents, with a minimum critical gelation concentration (CGC) of 0.5% *w*/*v* displayed in castor, heavy paraffin, jojoba, olive, linseed, sesame, and soybean oil, as well as 1.0% *w*/*v* displayed in the hazelnut, light paraffin oil, and PEG, respectively.

**Figure 1:**
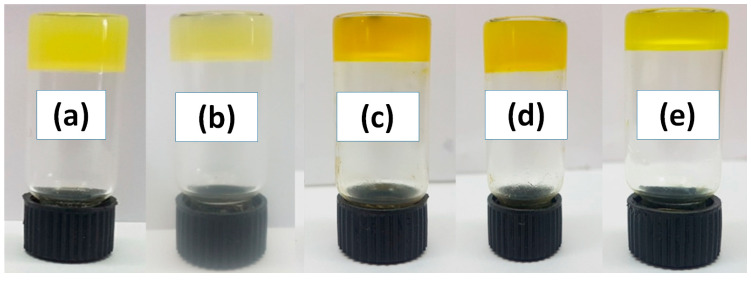
Images of gel formed by compounds **4a** and **4b**. (**a**) **4a** in soybean oil; (**b**) **4a** in sesame oil; (**c**) **4b** in soybean oil; (**d**) **4a** in sesame oil; and (**e**) **4b** in 1,2-dichlorobenzene.

When compared to vegetable and paraffin oils, a somewhat higher CGC is observed in alcohols and other organic solvents, such as ethylene glycol, lauryl alcohol, 1,2-dichlorobenzene, DMSO, and xylenes (Table 1). Representative images of the gels formed by compounds **4a** and **4b** in different solvents are presented in Figure 1. Further, gelator **4a,** with a kink in the alkyl chain, displayed gelation in glycerol with a CGC of 2.0%; whereas, the corresponding saturated compound (**4b**) did not form a gel. This suggested that the gelation property of low molecular weight gelators varied with their structure and the nature of the gelling solvent.

**Figure 2:**
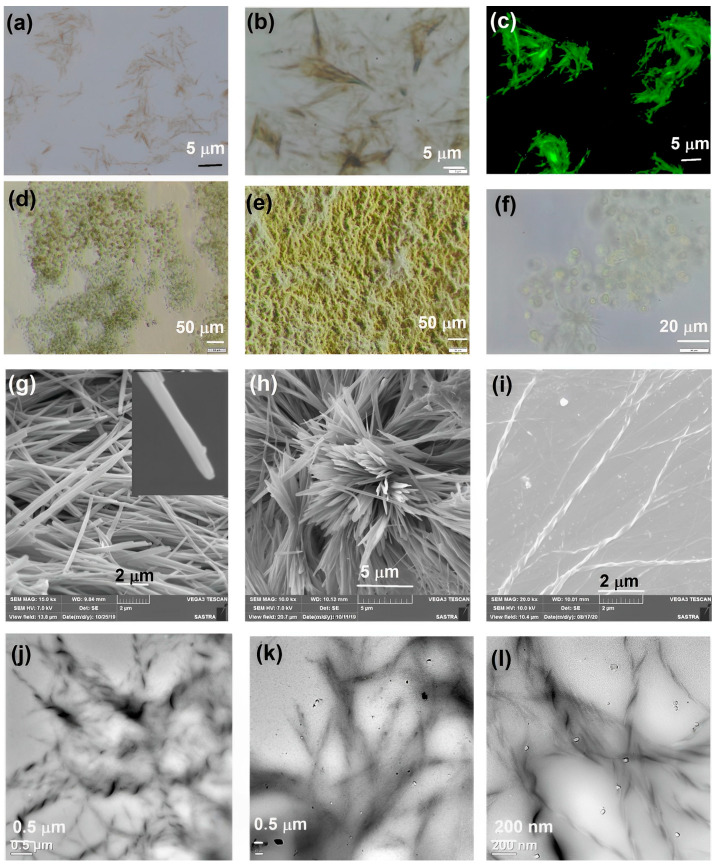
(**a**–**f**) Optical micrograph of gel formed by (**a**,**b**) **4a** in lauryl alcohol under white light and fluorescence light, respectively; (**c**) **4a** in PEG; (**d**) **4b** in soyabean oil; (**e**) **4b** in PEG; and (**f**) **4b** in ethylene glycol. SEM images of xerogel derived from (**g**) **4a** in lauryl alcohol; (**h**) **4a** in DMSO; and (**i**) **4b** in lauryl alcohol. (**j**–**l**) TEM images of oleogels formed by (**j**) **4a** in soybean oil; (**k**) **4a** in sesame oil; and (**l**) **4b** in olive oil.

Investigating the gelation ability of amphiphiles is not merely enough in the field of science and technology. During self-assembly, the way in which the individual molecule was associated to give highly ordered patterns, such as fibers, tubules, helices, lamellar, twisted fibers, micelles, and vesicles, which imparted unique properties to the assembled organic structure, was extremely important. The morphology of their self-assembled structures was obtained by optical microscopy, scanning electron microscope (SEM), and field emission transmission electron microscope (FETEM) analysis. The optical micrograph of gels formed by gelator **4a** in lauryl alcohol and PEG, as well as compound **4b** in soybean oil and PEG, revealed the existence of the micro or nanoscale organization of molecular aggregates with fibrillar morphology (Figure 2a–e). Compound **4b** in ethylene glycol displayed vesicular architecture, which formed a gel upon association.

A morphological investigation of gel using SEM revealed the existence of fibers of the widths 173–199 nm and 240–370 nm; twisted fibers of the width 97–242 nm were also identified by the gels obtained from **4a** in lauryl alcohol and DMSO, and those obtained from **4b** in lauryl alcohol, respectively (Figure 2g–i). A FETEM analysis of the oleogels formed by **4a** in soybean and sesame oil, and those formed by **4b** in olive oil, respectively, revealed the existence of twisted nanofibers (Figure 2j–l). It is worth mentioning that the non-covalent interactions, such as hydrogen bonding, π-π stacking, and van der Waals forces, were the main driving force for the molecular self-assembly; the change of gelation solvent drastically influenced the aggregation pattern [26,27].

**Figure 3:**
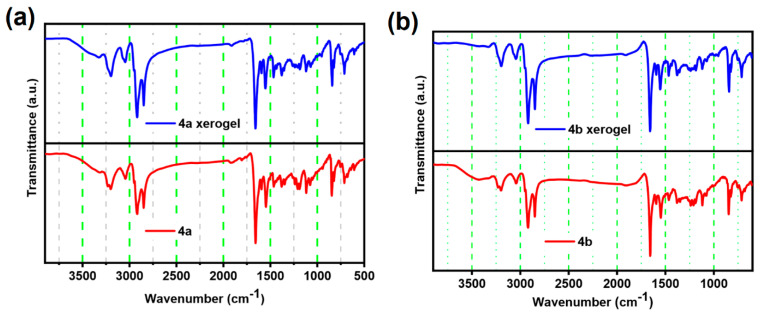
ATR-IR spectrum of the gelators (**a**) **4a** and (**b**) **4b** in the amorphous and xerogel states, respectively.

The existence of various intermolecular interactions in the molecular assembly of **4a** and **4b** was examined by attenuated total reflection-Fourier transform infrared spectroscopy (ATR-FTIR), X-ray diffraction (XRD), and emission studies. In particular, the ATR-FTIR studies provided a deep insight into the intermolecular interactions responsible for the self-organization that leads to the supramolecular architecture. The FTIR spectrum of **4a** and **4b** in amorphous and xerogel states is shown in Figure 3. Compound **4a** in the amorphous form displayed IR peaks of –OH, imine, amide I, amide II, and amide II while also showing overtones of aromatic C-H bending at 3199, 1657, 1629, 1547, 1348, 1920, 1806, and 1765 cm^−1^, respectively. Upon molecular assembly in xerogel, the peaks corresponded to amide I, amide II, and amide II, while also showing overtones of aromatic C-H bending shifted to 1622, 1553, 1354, 1912, 1788, and 1738 cm^−1^, respectively (Figure 3a). It is interesting to note that the peaks corresponding to –OH and imine did not display any shift, which directly implied the non-involvement of both groups in molecular self-assembly. We were very curious to investigate whether the saturated analog of **4a**, i.e., the saturation brought in the hydrophobic tail of the designed molecule (**4b**), could bring any change in self-assembly. The ATR-FTIR investigation of compound **4b** in the amorphous and xerogel states displayed a similar pattern of intermolecular interaction to that existing in **4a** without having any ambiguity (Figure 3b) [28,29]. The FTIR spectral studies clearly revealed the existence of intermolecular hydrogen bonding in the amide unit and π-π-stacking process in pyrene moiety. The imine unit and secondary hydroxyl group were not involved in the intermolecular interaction and the existing interactions were not affected by the unsaturation unit present in the hydrophobic part.

**Figure 4:**
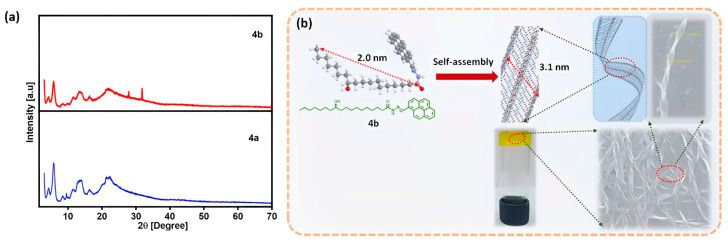
(**a**) XRD pattern of the xerogel formed from **4a** and **4b**; (**b**) Mechanism of molecular self-assembly of **4b**.

Having established the intermolecular interactions that exist in the fluorescent amphiphiles of **4a** and **4b**, it was mandatory to identify the molecular packing which influences the properties of advanced molecular-level materials. In order to visualize the molecular packing in gelators **4a** and **4b** within the gel matrix, a small-angle X-ray diffraction (SAXRD) method was used. The XRD patterns of the xerogel formed by **4a** in 1,2-dichlorobenzene displayed peaks at 2θ = 2.8, 4.31, 5.74, 7.91, 8.56, 9.79, 11.50, 13.1, 14.2, 16.25, 16.9, 19.0, 20.40, and 22.41, which corresponded to the interplanar spacing (d-spacing) of 3.1, 2.1, 1.5, 1.2, 1.03, 0.89, 0.76, 0.68, 0.62, 0.55, 0.52, 0.47, 0.44, and 0.40 nm, respectively. An analysis of XRD data clearly revealed that Bragg’s reflection of xerogel **4a** followed a progression ratio of 1:1/1.5:1/2:1/2.5:1/3:1/3.5:1/4:1/4.5:1/5:1/5.5:1/6:1/6.5:1/7:1/7.5, which was consistent with the regular arrangement of a gelator required to form a fibrous or twisted fibrous structure. It is worth mentioning that the xerogel obtained from **4b** also displayed a similar pattern of molecular arrangement with the broadening of the signal centered at 2θ = ~22°, as well as with two additional sharp peaks centered at 2θ = 27.8 and 31.7° with a d-spacing of 0.32 and 0.28 nm; this is also in line with the Bragg’s reflection progression (Figure 4). The XRD data clearly revealed the existence of molecular packing facilitated by an intermolecular interaction, such as intermolecular H-bonding in amide, the π-π stacking of the pyrene unit, and hydrophobic interactions. In order to predict the assembly pattern, the energy-minimized structures of **4a** and **4b** were obtained and the molecular length was calculated as 1.6 and 2.0 nm, respectively. The calculated end-to-end molecular length was much lower than the observed d-spacing value of 3.1 nm, clearly explaining the existence of a bilayer with an inter-digited fashion of arrangement [30].

**Figure 5:**
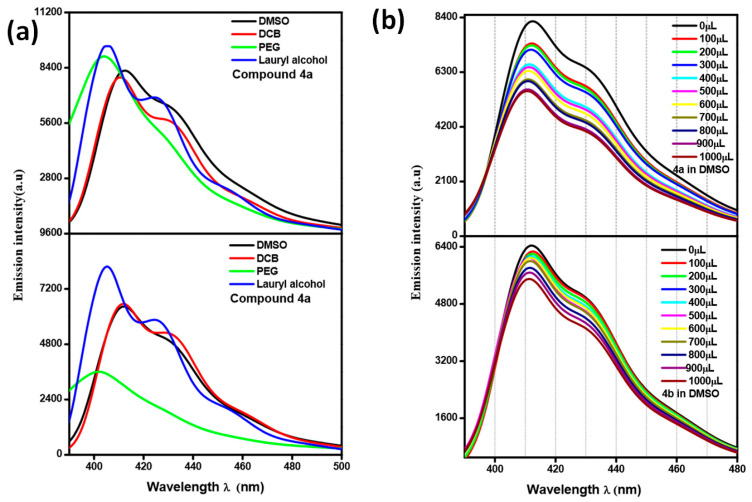

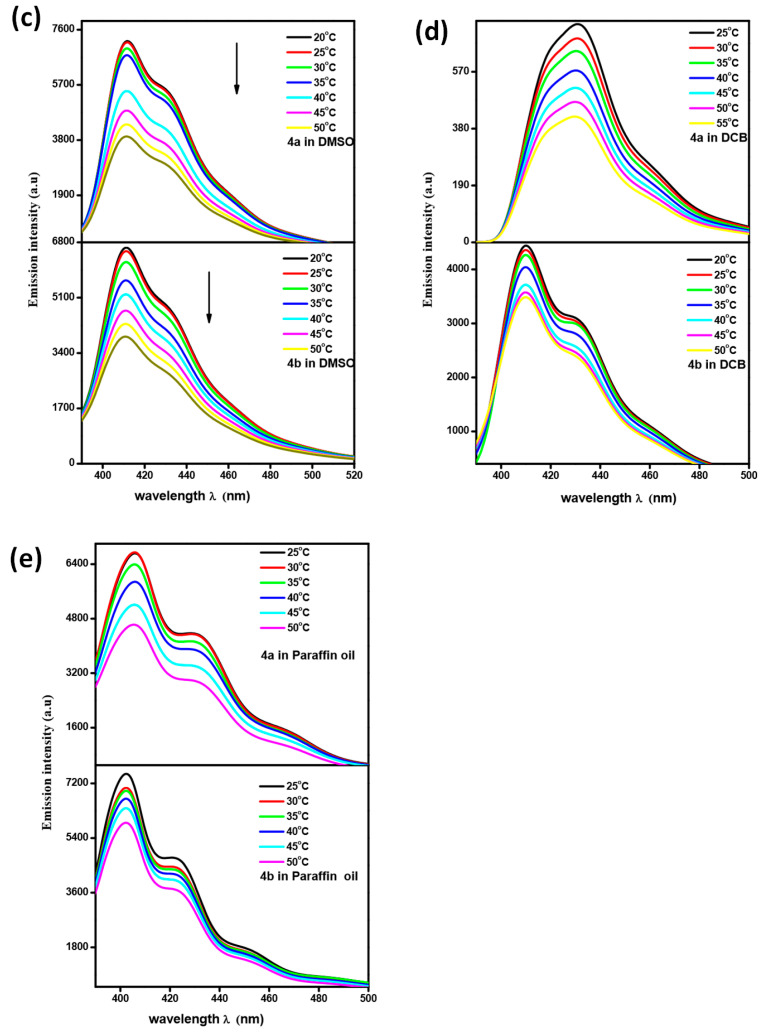
Emission spectra of (**a**) **4a** and **4b** in different solvents; (**b**) titration of a solution of **4a** and **4b** with DMSO; (**c**–**e**) variable temperature fluorescence spectra **4a** and **4b** in DMSO, DCB, and paraffin oil, respectively. In titration experiments, 2.0 mL of **4a**/**4b** (1 × 10^−5^ M) solution is used as the initial volume and the emission intensity is recorded for the addition of 100 μL of DMSO, followed by sonication.

Fluorescent gels, either as a standalone system or as light-emitting materials embedded in a gel system, can be used for sensing chemical or gas detection in minute quantities. The incorporation of highly conjugated moiety into the gelator skeleton is considered an important criterion to allowing electronic applications, such as sensors, optical, electrical memory, and display devices. In addition, the fluorescence emerging from such conjugated systems can also be used to act as an imaging agent in biomedicine. It is well known that during the process of molecular self-assembly, morphology is influenced by intermolecular and gelator-solvent interactions [31]. Even though FTIR and XRD studies could be able to provide deep insight into intermolecular interactions and molecular packing, emission studies further provide the details of gelator–solvent interactions and the existence of π-π staking [31,32]. To identify the response of the gelator in different gelling solvents, we recorded emission spectra for **4a** and **4b** in lauryl alcohol, PEG, DMSO, and dichlorobenzene (Figure 5a). Compounds **4a** and **4b** in DMSO at a 1 × 10^−5^ M concentration displayed an emission at 413 nm, which, upon sequential dilution with DMSO followed by sonication, displayed a hypsochromic shift (Figure 5b). When moving from the solution state to the assembled state, a bathochromic shift was observed (red shift), which is a consequence of the J-type π-π interaction that occurred in the assembled state and increased the molecular order. After obtaining exciting fluorescence results, we were curious to identify whether such J-type aggregation exists in the gel formed by the gelator in various solvents or not. The fluorescence spectra of **4a** and **4b,** recorded at variable temperatures in various solvents, such as DMSO, DCB, and paraffin oil, displayed a clear hypsochromic shift, which provides clear evidence for the disassembly of J-type aggregation (Figure 5c–e).

**Figure 6:**
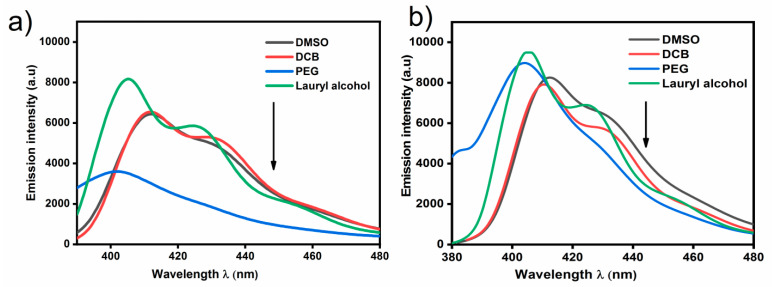
Emission spectra displaying a varying degree of solute-solvent interactions (**a**,**b**) **4a** and **4b** DMSO, 1,2-dicholorobenzene, polyethylene glycol, and Lauryl alcohol respectively.

In the field of supramolecular chemistry, the most challenging part is to understand the mechanical behavior of a gel because of the varying degrees of solute-solvent interactions, which are identified via emission studies (Figure 6). In particular, the flow behavior of gel is dependent on a three-dimensional fibrous network structure and the solute-solvent interactions. However, rheological studies are considered as a simple tool used to identify the processability of gels, especially when a function of stress applied or strain deformation is used, which is crucial for medical and technological applications.

**Figure 7:**
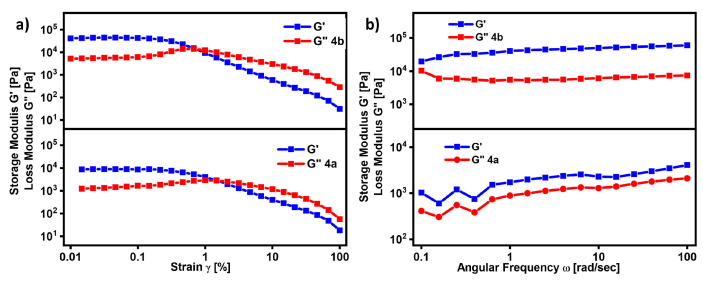
The dependence of G′ and G″ of gels formed from **4a**, **4b** in lauryl alcohol in (**a**) strain amplitude and (**b**) angular frequency.

**Figure 8:**
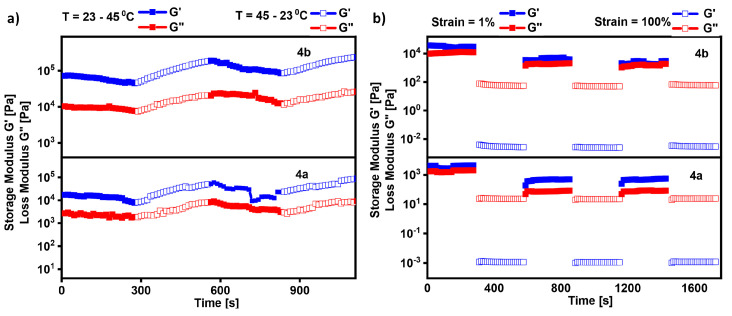
Rheological investigation of gels formed from **4a** and **4b** in lauryl alcohol (**a**) thermal processability using continuous temperature ramp-up and ramp-down experiments; (**b**) thixotropic behavior using continuous strain ramp-up and ramp-down experiments.

The mechanical properties were investigated by studying the viscoelastic properties of the gel formed by **4a** and **4b** in lauryl alcohol using a rheometer. Owing to the dependence of the gel deformation in response to stress or strain, with the nature and extent of intermolecular interactions causing supramolecular arrangements, flow characteristics are generally studied by measuring the strain amplitude sweep and angular frequency sweep necessity of gels with respect to storage modulus G′; this denotes the ability of the deformed gel to restore its original state (elastic nature) and loss modulus G″, which denote the flow characteristics of the gel [33]. Figure 7 shows the measurements of the strain amplitude and angular frequency dependence of the G′ and G″ of the gels formed from **4a** and **4b** in lauryl alcohol. Throughout the entire range of the frequency sweep measurements of these gels (**4a** and **4b**), the G′ was found to be greater than the G″ (Figure 7). This result revealed that these gels were stable and possessed a good tolerance to external forces. The amplitude sweep measurements clearly showed that both of these gels exhibited solid-like behavior up to a critical strain level γ_c_; at critical strain, a cross-over occurred between G′ and G″, after which both G′ and G″ decreased, indicating the flow characteristics of these gels. The γ_c_ for the gel formed from **4a** was 1.47% (G′ = G″ = 2802.7 Pa) whereas that for **4b** was 0.68% (G′ = G″ = 14,898 Pa). After identifying the potential of the gel to withstand external forces, the processability of the gels was examined by performing continuous temperature ramp-up and ramp-down experiments between 23 °C and 45 °C. Figure 8a indicates that these gels dissemble upon heating and regain the gel network upon cooling through reversible non-covalent interactions, confirming the thermo-reversible nature of these gels. Generally, the thixotropic nature of gels gives an idea about their ability to regenerate the structural integrity which was lost upon the application of varying strains to the gel [34,35,36]. Upon applying a constant strain of 100%, the gels from **4a** and **4b** became thinner; after reducing strain to 0.1%, the viscosity of the gels were reset to their initial state. We performed three cycles of continuous strain ramp-up and ramp-down experiments and observed the loss of structural integrity of the gel material with the increasing strain and the immediate recovery of the original state upon strain relaxation to 0.1% (Figure 8b) [32,37,38,39].

### 2.3. Sensing Measurements

**Figure 9:**
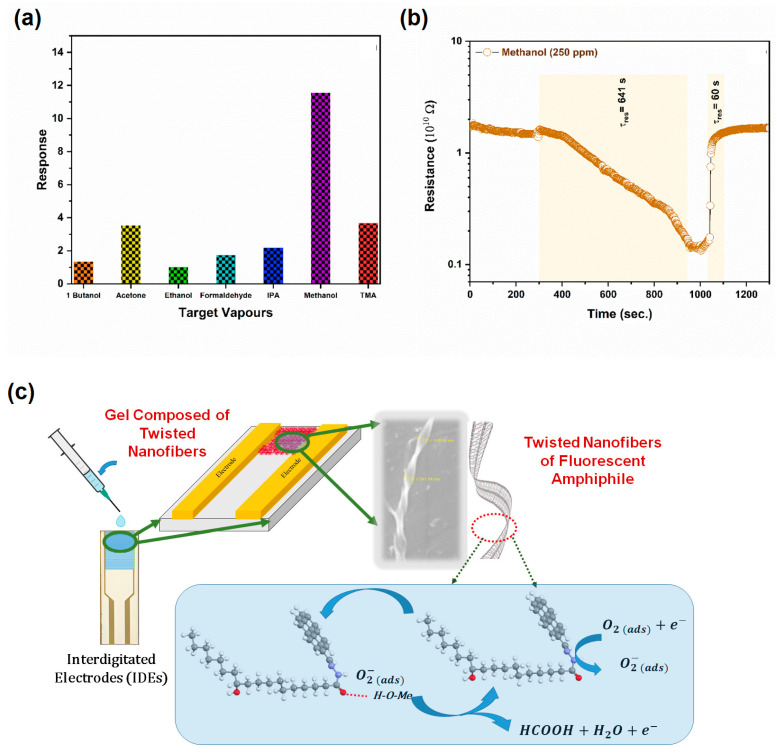
Sensing characteristics of electrode fabricated from the twisted fibers of **4a**. (**a**) Graph displaying selectivity towards methanol analyte; (**b**) Transient resistance response; and (**c**) Schematic representation of device fabrication and selective methanol sensing.

After establishing the molecular-level self-assembly mechanism, potential applications of the assembled materials were identified by performing sensing studies. Recently, the development of sensors to detect the presence of various harmful gases has been one of the most interesting areas of research. To investigate the gas-sensing behavior, sensing elements were prepared by drop casting the twisted fibers prepared from **4a** and **4b** on commercially available gold interdigitated electrodes (Ref. No.: P-IDEAU100, DropSens, Spain), followed by air-drying them at room temperature. The gas-sensing studies were performed using the custom-made gas-testing chamber reported on in our previous work [17,18,40]. The prepared electrode consisted of two interdigitated electrodes with two contact tracks. For sensors studies, twisted fibers derived from **4a** and **4b,** of concentration 1.0% *w*/*v*, were systematically coated on electrodes; sensing behavior was investigated using a high resistance electrometer (Keithley 6517B, USA) intergraded with a homemade chemical/gas-testing chamber [40]. In order to attain the precision of the fabricated sensor device, maintaining the gelator concentration of 1% *w*/*v* was crucial because the change in gelator concentration may have affected the porosity of the gel network, which would directly reflect on the sensor characteristics. Preliminary toxic gas-sensing studies revealed that the electrode decorated with the twisted fibers of **4a** was encouraging; hence, we selected the **4a**-coated electrode to use in investigating selectivity and sensitivity. The utility of assembled organic materials in vapor sensing is highly promising because of enhanced electron transport, facilitated ion transport, and versatile building blocks. Selectivity is one of the essential parameters for showing the ability of the device to identify the particular analyte, either in liquid or gaseous samples. Therefore, sensing elements fabricated from **4a** were examined in terms of different analytes and the sensing response was overserved via 250 ppm of 1-butanol, acetone, ethanol, formaldehyde, isopropanol (IPA), methanol, and trimethylamine (TMA). The sensor fabricated from **4a** displayed a selective response towards methanol over other target analytes (Figure 9a); this might be due to the diffusion and interaction of methanol, rather than the other target gases, as displayed above. It is worth mentioning that the fabricated sensor displayed a considerable response towards acetone and trimethylamine, rather than showing a response for the analogous molecules, such as ethanol and 1-butanol, which clearly revealed that in addition to pore aperture, the nature of the interaction was also an important phenomenon in the sensing response.

The sensing response of the twisted fiber derived from 4a can be calculated by using Equation (1),

S = R_a_/R_g_ (1)

where R_a_ and R_g_ are the surface resistance of the sensing element in ambient air and the analyte atmosphere, respectively.

Generally, the chemiresistive-based vapor/gas sensor mechanism follows the principle of change in chemi-resistance of the sensing element by an ad/desorption process of oxygen molecules via a charge transfer mechanism under various targeted analytes. In the initial stage, the baseline resistance (R_a_) of the sensing element was stabilized by exposing the sensing element to the ambient air atmosphere. In this stage, oxygen adsorption was taking place via the charge-transfer interaction between oxygen and the electron acceptor hydrazide moiety of **4a** [41], which led to the formation of a space-charge region with oxygen anions (O_2_^−^), in turn, increasing the surface resistance of the twisted fibers [Figure 9]. When the sensing element was exposed to reducing gas, such as methanol, the analyte reacted with the adsorbed oxygen ions, leading to the desorption of the adsorbed oxygen ions. The liberated electrons moved back to the conduction band of the sensing element. This desorption process resulted in an increased charge carrier concentration, in turn, decreasing the sensing element’s surface resistance (R_g_). Once the targeted vapors were removed from the sensing chamber, the surface resistance recovered to its baseline resistance due to the desorption of analytes. After completing the sensing studies of methanol, the twisted fibers of the **4a**-coated electrodes were kept in a vacuum hot-air oven set at a temperature of 80 °C for about 30 min. It is worth mentioning that, heating the electrode above 160 °C resulted in the decomposition of the twisted fibers. Figure 9b shows the time dependence of the twisted fibers derived from **4a** in terms of their surface resistance when exposed to a methanol analyte concentration of 250 ppm. The change in surface resistance was taken into account to calculate the sensing response of the fabricated electrode; the sensing mechanism towards methanol is given below in Equations (2)–(4) [42]. The electrode fabricated from the twisted fibers of **4a** displayed a selective response towards methanol over the other analytes. In order to ascertain the reproducibility of the sensing response (R_a_/R_g_), the twisted fibers derived from **4a** that were of a 1.0% *w*/*v* concentration were kept constant as we measured the response towards methanol in triplicate. The observed responses (R_a_/R_g_) were found to be within the range of 11 and 12 toward 250 ppm of methanol. It is worth mentioning that the change in the concentration of methanol influenced the sensing response of the device. The chemistry behind the selective response towards methanol was due to the diffusion and effective interaction with the acceptor bond sites of hydrazide moiety [43]. Based on the results, the device fabrication and methanol-sensing mechanism in twisted fibrous architecture are provided in Figure 9c.

(2) $$O_{2\;\left(\mathrm{atm}\right)}⇌O_{2\left(\mathrm{ads}\right)}$$

(3) $$O_{2\;\left(\mathrm{ads}\right)}+e^{-}⇌O_{2\;\left(\mathrm{ads}\right)}^{-}$$

(4) $${\mathrm{CH}}_{3}\mathrm{OH}+O_{2}^{-}\to \mathrm{HCOOH}+H_{2}O+e^{-}$$

## 3. Conclusions

In summary, we developed a simple protocol for the generation of a fluorescent π-gelator from methyl ricinoleate in good yield. The supramolecular self-assembly of π-gelator in a wide range of solvents furnished twisted-fiber and fibrillar bundle-like architecture. The morphological analysis of the self-assembled structures using optical microscopy, SEM, and TEM revealed the existence of different patterns of arrangement with respect to the assembly environment. The bottom-up assembly mechanisms of π-gelator involving various types of intermolecular interactions were identified by FTIR, XRD, and emission studies; a suitable assembly mechanism has been proposed. The stability and processability of the twisted-fiber and fibrillar bundle-like architecture in gels were investigated using rheological measurements. Gels derived from **4a** and **4b** displayed a good tolerance towards external forces, thermal processability, and thixotropic behavior; hence, the gel could be easily fabricated into the desired material. The highly-tunable properties displayed by the assembled material with the integration of the π-unit expanded the scope of utilizing it in electronic and sensor industries. The determination of the sensing features of the twisted-fiber-decorated electrodes, using a high resistance electrometer intergraded with a homemade chemical/gas testing chamber, disclosed the potential use of twisted fibers for sensing 250 ppm of 1-butanol, acetone, ethanol, formaldehyde, isopropanol, methanol, and trimethylamine, with a selective response towards methanol over the other target analytes. The thermo-reversibility, long-term stability of gel material under atmospheric conditions, and preliminary sensor studies on twisted fibers guaranteed the robustness and reliability of the gels in gas-sensor technology.

## 4. Experimental Section

### 4.1. Materials and Methods

All reagents and solvents required for the synthesis of compounds **4a** and **4b** were procured from TCI (India), Sigma Aldrich (India), Merck (India), Alfa Aesar (Mumbai, India), and Avra (Hyderabad, India) and were used as such, without any distillation/purification. For compound purification and recrystallization, LR-grade solvents were employed. AR-grade solvents were used for the gelation studies. The pre-coated Merck silica gel plates were used to monitor the reaction progress by Thin-layer chromatography (TLC) and we visualized the spots using any one, or a combination, of the visualizing agents, such as UV detection, KMnO_4_, p-anisaldehyde stain spray, and molecular iodine.

In addition, the ^1^H and ^13^C NMR spectra were recorded on a Bruker Avance 300 MHz instrument, either in CDCl_3_ or CDCl_3_, with a few drops of DMSO-d_6_ at room temperature. Chemical shifts (δ) were reported in parts per million (ppm), with reference to the internal standard TMS, and coupling constants (J) were given in Hz. Proton multiplicity was assigned using the following abbreviations: singlet (s), doublet (d), triplet (t), quartet (q), and multiplet (m). High-resolution MS analysis was performed on an Agilent 6520 Q-TOF instrument by dissolving the solid sample in methanol.

To obtain the morphology of the gel, optical microscopy, scanning electron microscopy (SEM), and high-resolution transmission electron microscopy (HRTEM) were carried out using a Carl Zeiss AXIO ScopeA1 fluorescent/phase contrast microscope, a JEOL JSM-6701F ultrahigh resolution field emission scanning electron microscope, and a JEOL JEM 2100 F HRTEM, respectively. The XRD measurements were taken by keeping a small portion of the xerogel in the X pert-PRO Diffractometer system. The UV/vis spectra were recorded on a Thermo Scientific Evolution 220 UV/visible spectrophotometer. The spectra were recorded in the constant mode between 200 and 700 nm, with a wavelength increment of 1 nm and a bandwidth of 1 nm. the emission spectra were measured on a JASCO spectrofluorometer FP8200.

### 4.2. Gelation Studies

The gelation ability of the target compounds **4a** and **4b** were investigated by the “stable to inversion method” in a diverse range of organic solvents, such as DMSO, DMF, dichlorobenzene, xylene, glycerol, lauryl alcohol, polyethylene glycol, etc., and oils, such as soybean, olive, jojoba, hazelnut, castor, linseed, light paraffin, heavy paraffin, etc. The procedure for the self-assembly of the amphiphiles to form the gel was as follows: an appropriate amount of solvent/oil was added to the calculated quantity of the gelator, taken in a vial, and sealed. First, uniform dispersion of the compound in a suitable solvent was achieved by sonication. The complete dissolution of the gelator occurred by heating the uniformly dispersed solution; then, the subsequent cooling to room temperature resulted in the gel formation. The stage in which there was no gravitational flow in the inverted vial denoted the gelation, “G”. At the end of the gelation test, if it remained as a solution, it was referred to as “S”; if it remained as a precipitate, it was indicated by a “P”. If the dispersed gelator did not dissolve upon heating, it was referred to as insoluble (I).

### 4.3. Rheological Measurements

The visco-elastic characteristics of the gel derived from compounds **4a** and **4b** were investigated by carrying out the rheological measurements at 23 °C using a stress-controlled rheometer (Anton Paar Modular Compact Rheometer 302) furnished with a 25 mm diameter steel-coated parallel-plate geometry. Rheological measurements for the gels were recorded by placing the gel sample over the parallel plate with a 1 mm gap between the plates before subsequently trimming the excess gel.

### 4.4. Synthesis

#### 4.4.1. Synthesis of Compound **1b**

After obtaining a methanolic solution of methyl ricinoleate, **1a** (1.0 mmol) was added Pd/C (10%) and stirred under a hydrogen atmosphere for 18 h. After the completion of the reaction, as identified by TLC, the resulting mixture was filtered over a celite bed and washed with methanol. The solvent was removed under a vacuum to obtain pure product **1b** [16].

Compound **1b**. Isolated as a pale white solid; yield: 94%: 1H NMR (300 MHz, CDCl_3_): δ = 3.60 (s, 3H, –COOCH_3_), 3.55–3.44 (m, 1H–CH–) 2.34–2.20 (m, 2H, –CH_2_), 1.57–1.48 (m, 4H, –CH_2_–), 1.37–1.16 (m, 22H, –CH_2_), 0.81 (t, J = 6.0 Hz, 3H, –CH_2_CH_3_); 13C NMR (75 MHz, CDCl_3_): δ = 174.3, 71.9, 51.4, 42.8, 37.5, 34.1, 31.9, 31.6, 29.7, 29.4, 29.2, 29.1, 28.9, 25.6, 24.9, 23.9, 22.7, 22.6,14.1.

#### 4.4.2. Synthesis of Compound **2**

After obtaining an ethanolic solution of methyl ester **1a** or **1b** (1.0 mmol), hydrazine hydrate (1.2 mmol) was added and refluxed with stirring for 4 h. After confirming the completion of the reaction using TLC, the reaction mixture was cooled to room temperature and the precipitated hydrazide (**2a** or **2b)** was filtered, dried, and recrystallized in methanol.

Compound **2a**. Isolated as a pale yellow solid; yield: 91%: ^1^H NMR (300 MHz, CDCl_3_): δ = 6.79 (s (br), 1H, –NH), 5.56–5.36 (m, 2H, Alk-H), 3.92 (s (br), 2H, –NH2), 3.62–3.56 (m, 1H, –CH–) 2.23–2.03 (m, 4H, –CH_2_–), 1.67–1.59 (m, 2H, –CH_2_–), 1.45–1.39 (m, 2H– CH_2_–), 1.34–1.25 (m, 18H, –CH_2_–), 0.88 (t, J = 6.0 Hz, 3H, CH_3_); ^13^C NMR (75 MHz, CDCl_3_ + DMSO–d_6_): δ = 178.0, 136.1, 131.1, 75.5, 42.3, 41.7, 40.2, 38.9, 36.6, 34.5, 34.3, 34.2, 34.0, 33.8, 32.1, 30.4, 27.3, 19.0.

Compound **2b**. Isolated as a pale white solid; yield: 90%: ^1^H NMR (300 MHz, CDCl_3_): δ = 6.84 (s (br), 1H, –NH), 4.0–3.50 (m, 3H, –CH– and –NH_2_), 2.33–2.10 (m, 4H, –CH_2_–), 1.67–1.58 (m, 2H, –CH_2_–), 1.49–1.28 (m, 24H, –CH_2_–), 0.88 (t, J = 6.0 Hz, 3H, –CH_3_); ^13^C NMR (75 MHz, CDCl_3_ + DMSO-d_6_): δ = 178.1, 75.5, 56.1, 42.3, 38.7, 36.6, 34.5, 34.3, 34.2, 34.1, 34.0, 33.9, 33.8, 30.5, 30.4, 27.3, 19.0.

#### 4.4.3. Synthesis of Compound **4**

After obtaining the methanolic solution of hydrazides **2** (1.0 mmol) and triethylamine (1.5 mmol), 1-pyrenecarboxaldehyde (1.0 mmol) was added and refluxed with stirring for 6 h. The progress of the reaction was monitored using TLC. After confirming the completion of the reaction, the reaction mixture was cooled to room temperature and the yellow precipitate formed was filtered and dried under a vacuum.

Alternatively, compound **4** could be synthesized in one step, even without the use of the additional organic base, triethylamine. The one-pot synthetic procedure is as follows: a methanolic solution of methyl ester **1a** or **1b** (1.0 mmol) was obtained and hydrazine hydrate (1.2 mmol) was added and refluxed with stirring for 4 h. Without isolating the hydrazides (**2**), 1-pyrenecarboxaldehyde (1.0 mmol) was added and refluxed with stirring for 1 h. The progress of the reaction was monitored using TLC. After confirming the completion of the reaction, the reaction mixture was cooled to room temperature and the yellow precipitate formed was filtered and dried under a vacuum.

Compound **4a**. Isolated as yellow solid; yield: 72%: ^1^H NMR (600 MHz, CDCl₃): δ = 9.47 (s, 1H, –NH), 8.74 (s, 1H, –CH=N), 8.69 (d, J = 12.0 Hz, 1H, Ar-H), 8.47 (d, J = 12.0 Hz, 1H, Ar-H), 8.25–8.00 (m, 7H, Ar-H), 5.59–5.50 (m, 1H, –CH=CH–), 5.49–5.32 (m, 1H, –CH=CH–), 3.60–3.56 (m, 1H, –CH–), 2.91 (t, J = 6.0 Hz, 2H, –CH_2_–), 2.18 (t, J = 6.0 Hz, 1H, –CH_2_–), 2.06–2.03 (m, 1H, –CH_2_–), 1.87–1.81 (m, 2H, –CH_2_–), 1.65–1.59 (m, 2H, –CH_2_–), 1.51–1.25 (m, 18H, –CH_2_–) 0.86 (t, J = 6.0 Hz, 3H, –CH_3_)); ^13^C NMR (150 MHz, CDCl_3_): δ = 176.8, 142.6, 133.4, 132.5, 131.4, 130.7, 129.2, 128.7, 128.5, 127.5, 126.6, 126.3, 126.0, 125.7, 125.3, 125.1, 124.7, 122.5, 71.6, 37.6, 36.9, 35.4, 33.1, 31.9, 29.8, 29.7, 29.6, 29.4, 29.3, 29.2, 29.1, 27.5, 27.4, 25.8, 25.7, 25.0, 22.7, 14.2.

Compound **4b**. Isolated as yellow solid; yield: 70%: ^1^H NMR (300 MHz, DMSO-d_6_): δ = 9.28 (s, 1H, –NH), 8.98 (s, 1H, –CH=N), 8.72–8.51 (m, 1H, Ar-H), 8.49 (m, 1H, Ar-H), 8.25–8.0 (m, 7H, Ar-H), 3.50 (s (br), 1H, –OH), 3.48–3.32 (m, 1H, –CH–), 2.85 (t, J = 7.5 Hz, 1H, –CH_2_–), 2.39–2.34 (m, 1H, –CH_2_–), 1.81–1.76 (m, 2H, –CH_2_–), 1.50–1.20 (m, 26H, –CH_2_–), 0.86 (t, J = 6.0 Hz, 3H, –CH_3_)); 13C NMR (75 MHz, CDCl_3_ + DMSO-d_6_): δ = 174.5, 146.4, 136.9, 136.7, 136.0, 133.5, 133.4, 133.0, 132.3, 132.2, 131.2, 130.7, 130.4, 129.9, 129.6,129.5,129.2, 127.4, 75.3, 42.4, 39.9, 37.6, 36.6, 34.5, 34.3, 34.2, 30.5, 29.6, 27.4, 19.0. HRMS (ESI, *m*/*z*): [M + H]^+^ calculated for C_35_H_46_N_2_O_2_: 527.3632; found: 527.3664.

### 4.5. X-ray Diffraction and Molecular Modelling

For SAXRD analysis, a small portion of xerogel was placed down and a diffraction pattern was obtained using a BRUKER-binary V3 diffractometer system. ChemDraw Professional 16 and Mercury—The Cambridge Crystallographic Data Centre (CCDC) software was used to obtain the MM2 energy-minimized diagram and 3D structure visualization, respectively.

### 4.6. Sensor Measurements

Sensing characteristics were investigated using homemade chemical/gas-testing chamber44, which was integrated with a high-resistance electrometer (Keithley 6517 B, Beaverton, OR, USA). The sensing studies were performed by coating the twisted fibers of **4a** onto the electrodes and drying them in a vacuum hot-air oven at 80 °C for about 30 min. After the initial screening, a response toward a specific analyte was performed in triplicate.

## Figures and Tables

**Table 1:** Gelation studies of compounds **4a** and **4b** in various solvents/oils.

S.No.	Solvent/Vegetable Oils	Observation (CGC in % *w*/*v*)	
		4a	4b
1	Castor oil	G(0.5)	G(0.5)
2	Eucalyptus oil	S	S
3	Hazelnut oil	G(1)	G(1)
4	Heavy paraffin oil	G(0.5)	G(0.5)
5	Jojoba oil	G(0.5)	G(0.5)
6	Light paraffin oil	G(1)	G(1)
7	Linseed oil	G(0.5)	G(0.5)
8	Olive oil	G(0.5)	G(0.5)
9	Sesame oil	G(0.5)	G(0.5)
10	Soybean oil	G(0.5)	G(0.5)
11	DMF	S	S
12	H_2_O	I	I
13	DMSO	G(1.5)	G(1.5)
14	Ethyl acetate	I	I
15	Cyclohexane	I	I
16	Xylene	G(2)	G(2)
17	Glycerol	G(2)	S
18	Ethylene Glycol	G(1.5)	G(1.5)
19	Poly (ethylene glycol)	G(1)	G(1)
20	Methanol	I	I
21	1,2-dichlorobenzene	G(1.5)	G(1.5)
22	Lauryl alcohol	G(1.5)	G(1.5)

## Data Availability

Complete experimental data is provided in the article and supporting information, and no new data were created.

## Supplementary Materials

The following supporting information can be downloaded at: https://www.mdpi.com/article/10.3390/gels9060442/s1, NMR, and Mass spectra. Supporting information: Gelation studies, NMR spectra, Mass spectra, and UV-Vis spectra.
