# Supplementary material for: Self-Assembling Nanoarchitectonics of Twisted Nanofibers of Fluorescent Amphiphiles as Chemo-Resistive Sensor for Methanol Detection
Source: Gels. 2023 May 26;9(6):442. doi: 10.3390/gels9060442 (PMC10298205; doi:10.3390/gels9060442)
Supplement: Supplementary file 1 [file gels-09-00442-s001.zip › gels-2377643-supplementary.pdf]

Supplementary Materials

Self-Assembling Nanoarchitectonics of Twisted Nanofibers of Fluorescent Amphiphiles as Chemo-Resistive Sensor for Methanol Detection

Vandana Singh [1], Ayyapillai Thamizhanban [1], Krishnamoorthy Lalitha [1], Dinesh Kumar Subbiah [2], Arun Kumar Rachamalla [3], Vara Prasad Rebaka [3], Tohira Banoo [3], Yogendra Kumar [3], Vellaisamy Sridharan [4], Asrar Ahmad [5], C. Uma Maheswari [1], John Bosco Balaguru Rayappan [2], Azmat Ali Khan [6,*] and Subbiah Nagarajan [1,3,*]

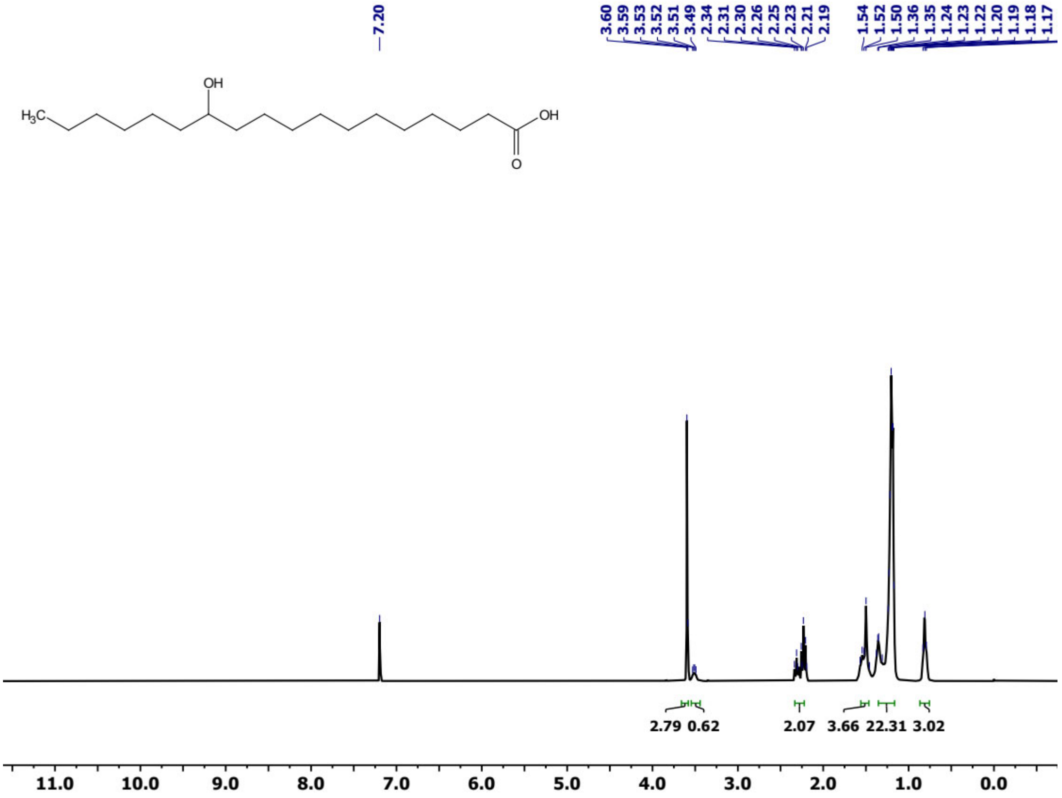

Figure S1. ^{1}H NMR of the compound, **1b** in CDCl$_3$.

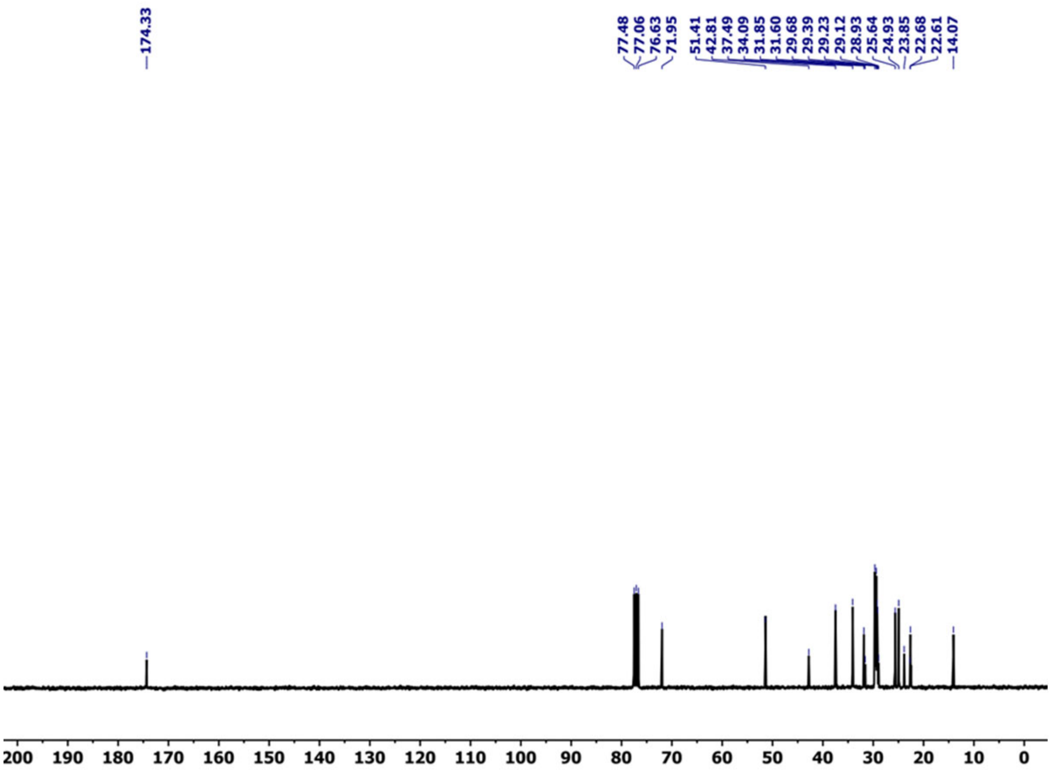

Figure S2. ^{13}C NMR of the compound, **1b** in $CDCl_3$.

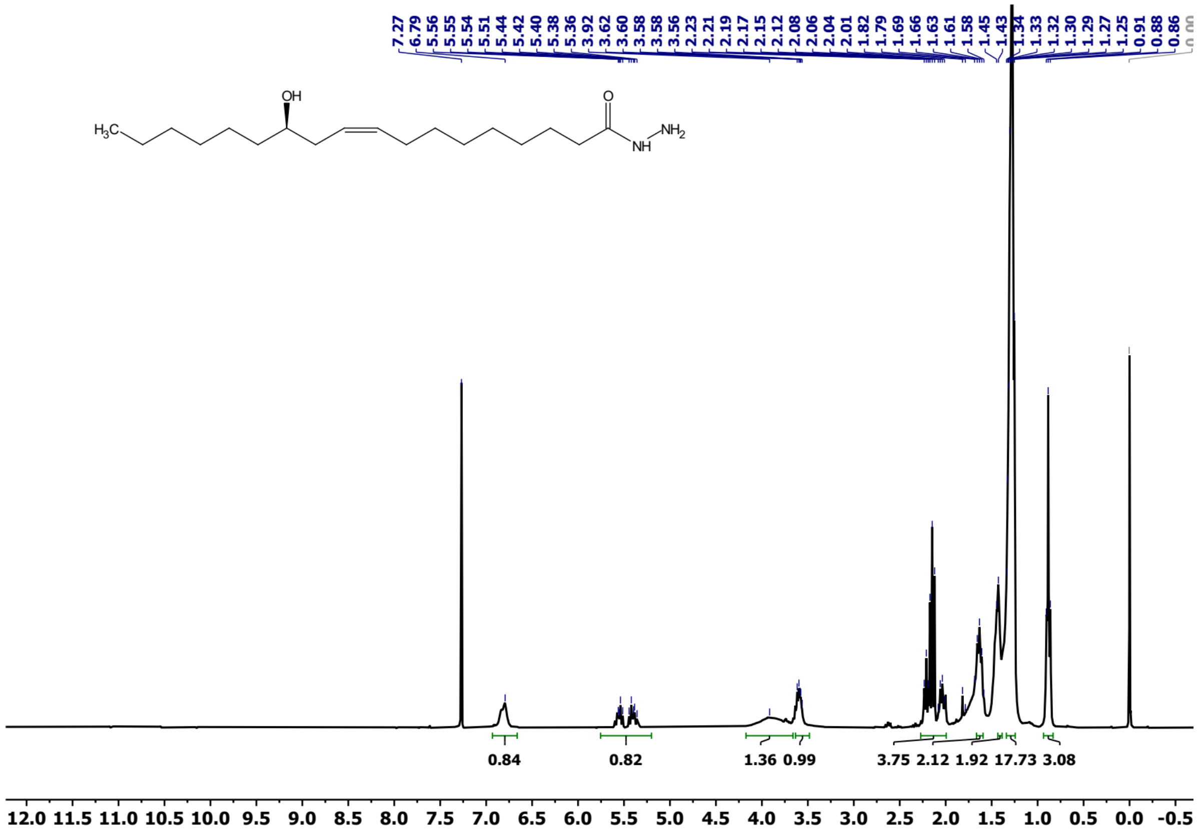

Figure S3. ^{1}H NMR of the compound, **2a** in $CDCl_3$.

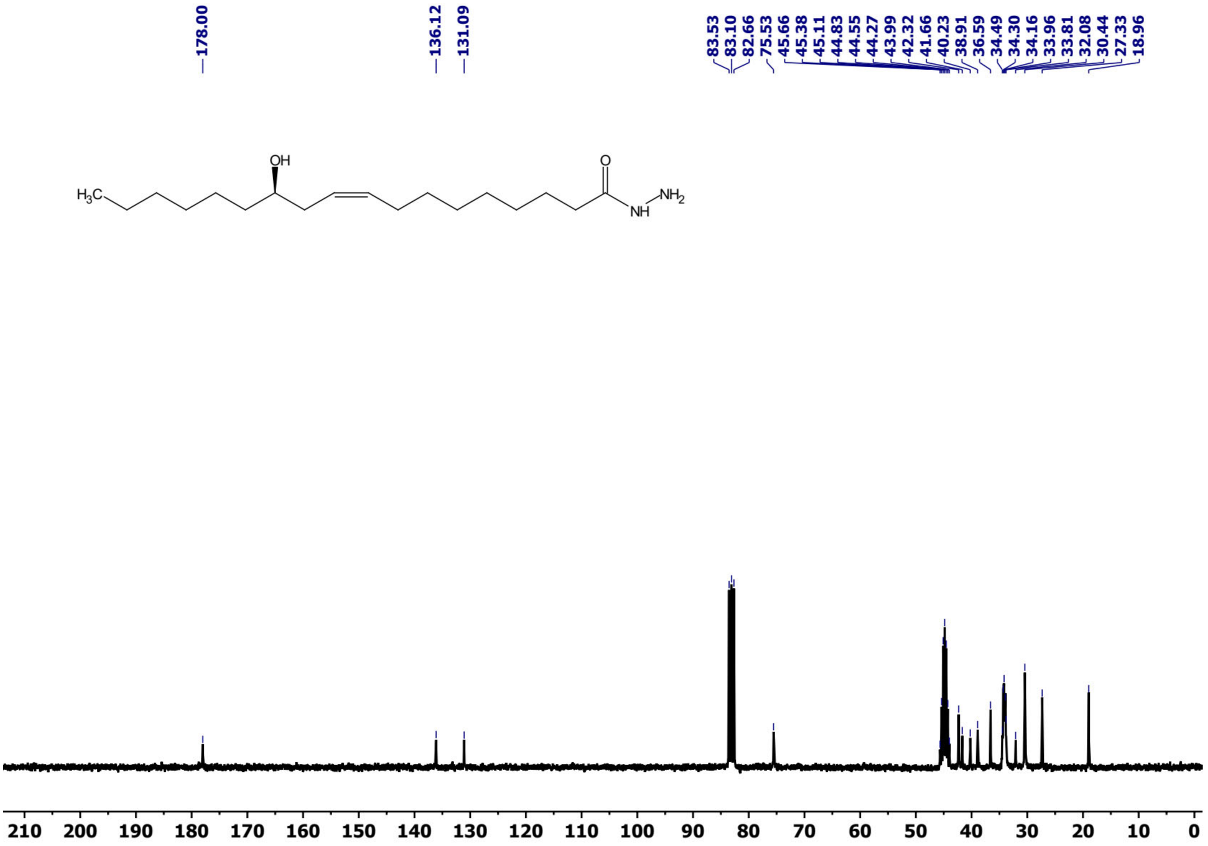

Figure S4. ^{13}C NMR of the compound, **2a** in $CDCl_3$+DMSO-d_6.

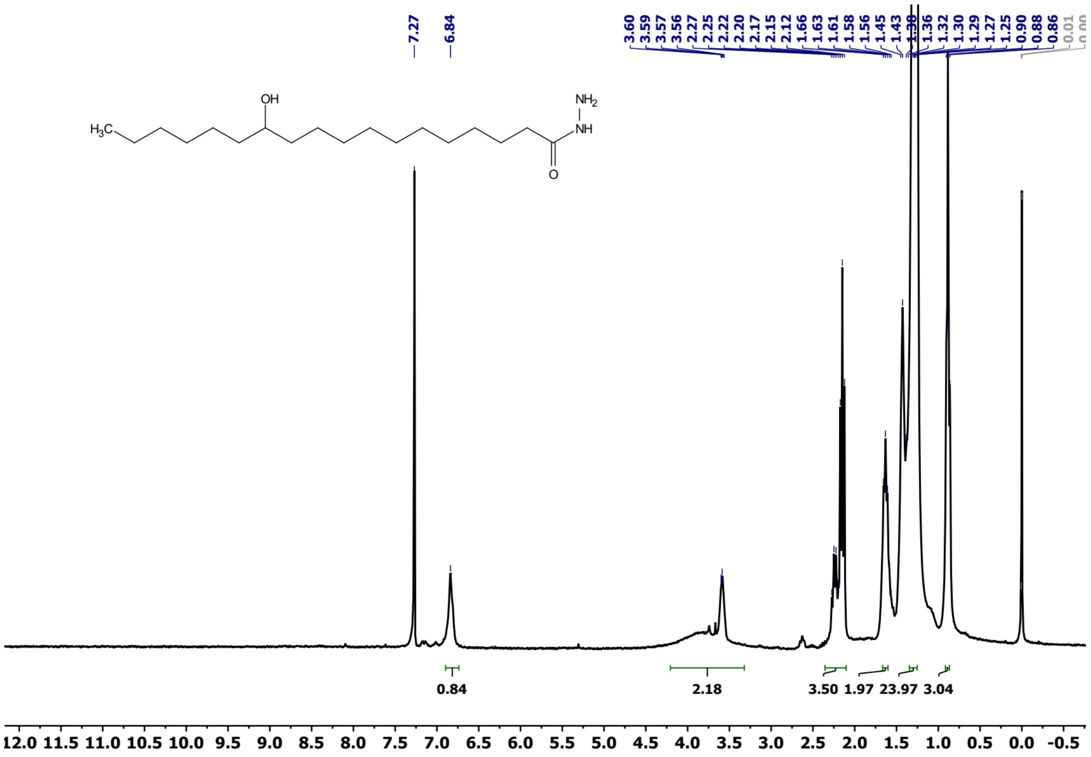

Figure S5. ^{1}H NMR of the compound, **2b** in $CDCl_3$.

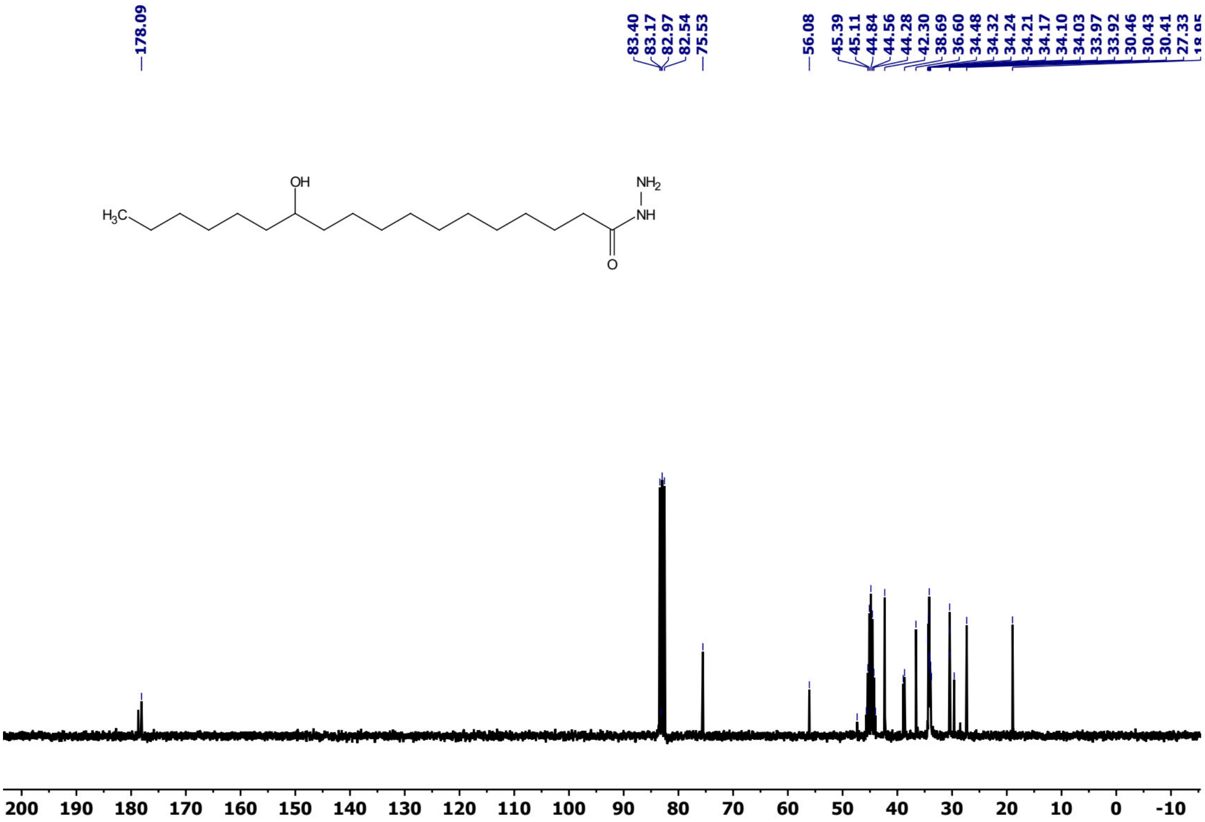

Figure S6. ^{13}C NMR of compound, **2b** in $CDCl_3$+DMSO-d_6.

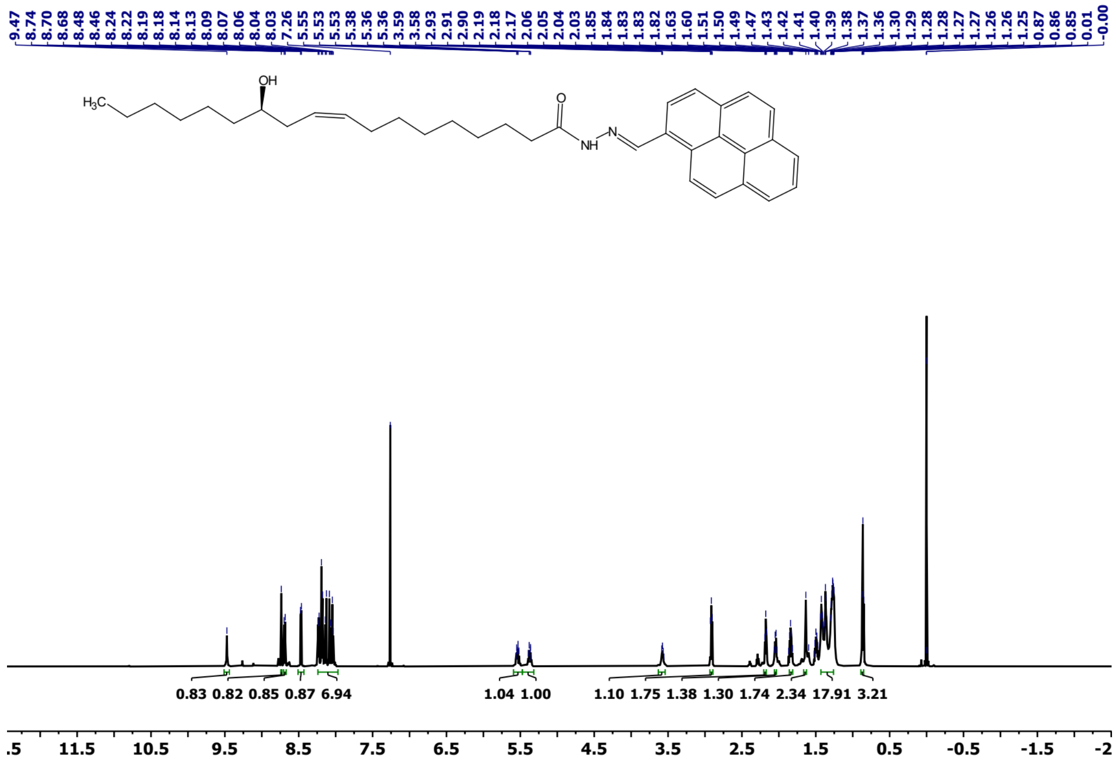

Figure S7. ^{1}H NMR of the compound, **4a** in $CDCl_3$.

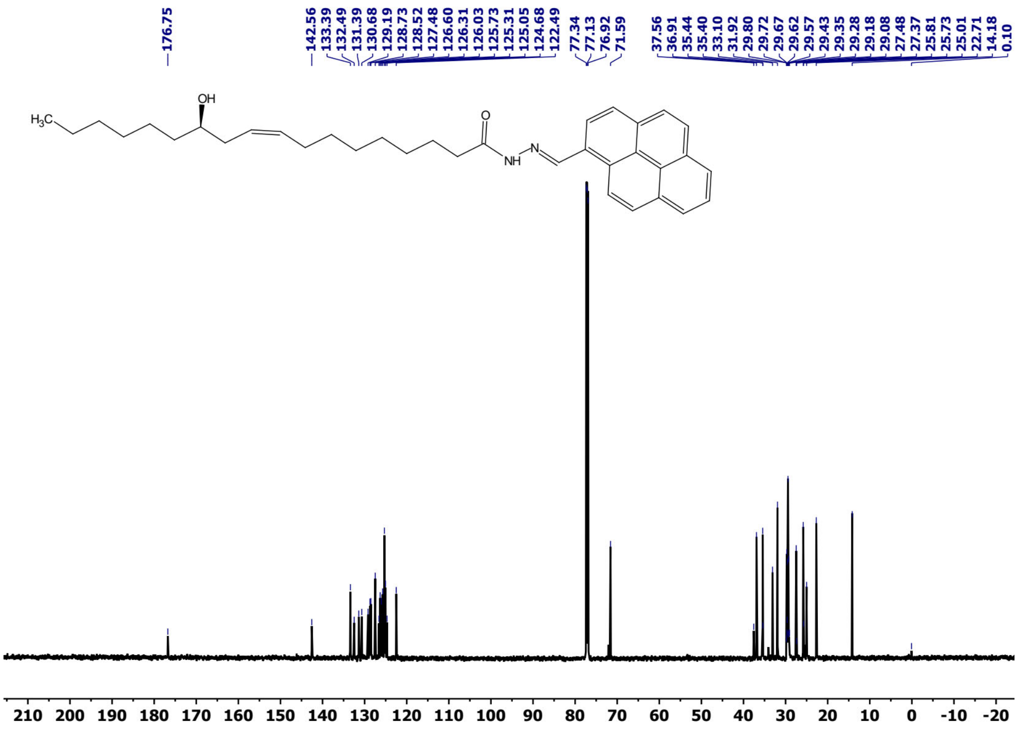

Figure S8. ^{13}C NMR of the compound, **4a** in $CDCl_3$.

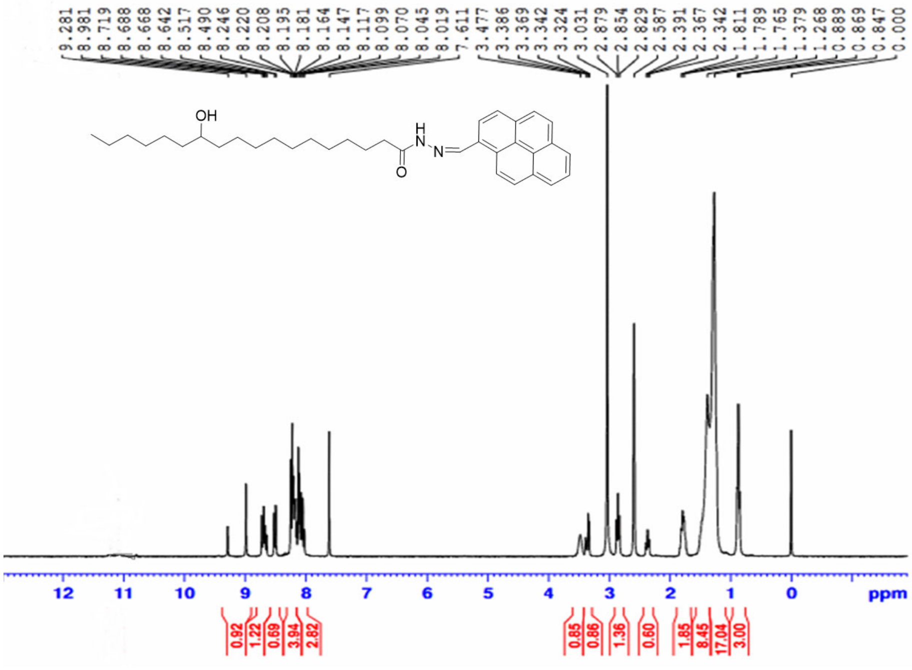

Figure S9. ^{1}H NMR of compound, **4b** in DMSO-d_6.

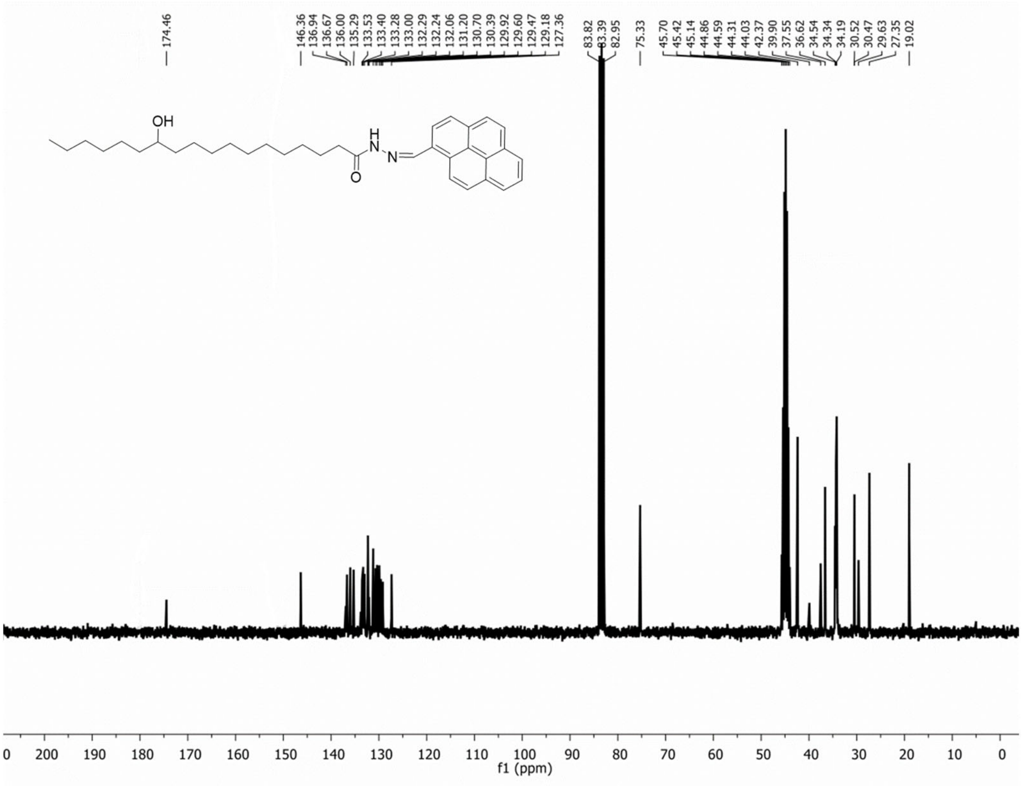

Figure S10. [13] C NMR of compound, **4b** in DMSO-d_6.

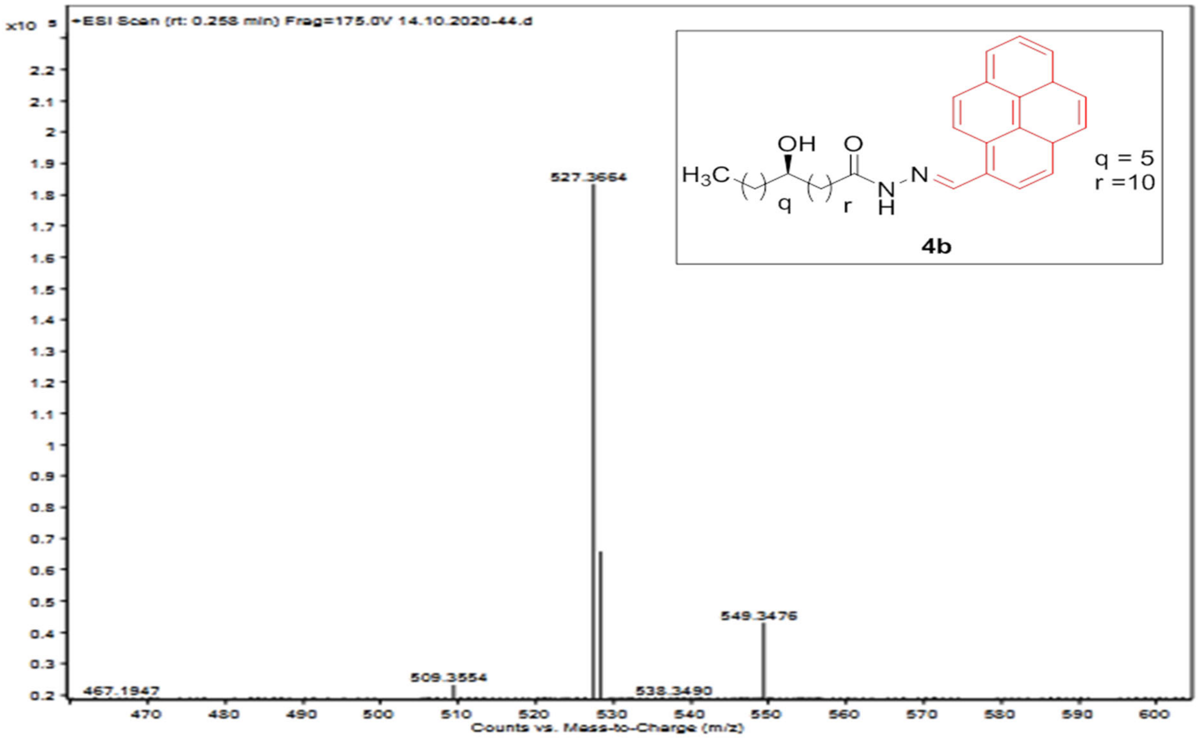

Figure S11. ESI-MS spectra of compound **4b.**
